# Medical ethnobotany of the Albanian Alps in Kosovo

**DOI:** 10.1186/1746-4269-8-6

**Published:** 2012-01-28

**Authors:** Behxhet Mustafa, Avni Hajdari, Feriz Krasniqi, Esat Hoxha, Hatixhe Ademi, Cassandra L Quave, Andrea Pieroni

**Affiliations:** 1 Department of Biology, University of Prishtina, Mother Teresa, Prishtinë, Kosovo; 2 Kosovo Academy of Sciences and Arts, Agim Ramadani, Prishtinë, Kosovo; 3 Center for the Study of Human Health, Emory University, 550 Asbury Circle, Candler Library 107E, Atlanta, GA 30322 (USA; 4 University of Gastronomic Sciences, Piazza Vittorio Emanuele 9, I-12060 Pollenzo (Italy

**Keywords:** Albanian Alps, Ethnobotany, Traditional Medicine, Kosovo, Medicinal plants

## Abstract

**Background:**

Ethnobotanical studies are crucial in South-Eastern Europe for fostering local development and also for investigating the dynamics of Traditional Environmental Knowledge (TEK) related to plants in one of the most crucial European hotspots for biocultural diversity. The current medico-ethnobotanical survey was conducted in rural alpine communities in Kosovo. The aims of the study were twofold: 1) to document the state of TEK of medicinal plants in these communities; 2) to compare these findings with that of similar field studies previously conducted among local populations inhabiting the Montenegrin and Albanian side of the same Alpine range.

**Methods:**

Field research was conducted in 36 villages on the Kosovar side of the Albanian Alps. Snowball sampling techniques were used to recruit 91 elderly informants (≥ 50 years-old) for participation in semi-structured interviews and structured surveys regarding the use of the local flora for medicinal and food purposes. Standard ethnobotanical methods were employed and prior informed consent was obtained for all study participants.

**Results and Conclusion:**

The uses of 98 plants species belonging to 39 families were recorded; the most quoted botanical families were Rosaceae, Asteraceae, and Lamiaceae. Mainly decoctions and infusions were quoted as folk medicinal preparations and the most common uses referred to gastrointestinal and respiratory disorders, as well as illnesses of the uro-genital system. Among the most uncommon medicinal taxa quoted by the informants, C*arduus nutans *L., *Echinops bannaticus *Rochel ex Schrad., and *Orlaya grandiflora *Hoffm. may merit phytochemical and phytopharmacological investigations.

Comparison of the data with other ethnobotanical field studies recently conducted on the Albanian and Montenegrin sides of the same Alps has shown a remarkable link between the medical ethnobotany of Montenegrin and Kosovar side of the Albanian Alps. Moreover, folk uses of the most quoted wild medicinal taxa recorded in Kosovo often include those recorded both in Albania and in Montenegro, thus suggesting a hybrid character of the Kosovar local plant knowledge. This may be also explained with the fact that Montenegro and Kosovo, despite their differences in the ethnic composition, have shared a common history during the last Century.

## Background

Ethnobotanical studies in South-Eastern Europe are seen as a crucial initial step for local rural development based on eco-tourism, small-scale trade of local medicinal plants, high-quality local foods, eco-museums, and community-based bio-conservation strategies [1].

However, this region is also considered very special for conducting studies having a human ecological focus, since it represents a unique hotspot of biological and cultural diversity in Europe, thus allowing cross-cultural comparisons of traditional environmental knowledge (TEK) concerning medicinal plants. In very recent years, the Western Balkans have been the focus of a remarkable number of ethnobotanical studies [2-9], mainly focused on mountainous communities [10-15].

In this study, we investigated the Kosovo side of the Albanian Alps (in Albanian known as Bjeshkët e Nemuna or Alpet Shqipëtare; in Serbo-Croatian known as Prokletije), which extends within a triangle among the Dinaric Mountains in the North-West, the Sharri (Šar) Mountains in the South-East and the Rhodope Mountains in the East and North-East. This covers a very pristine, and sometimes, remote area of ca. 3,500 km^2^, which is geo-politically divided among the sovereign states of Albania, Kosovo, and Montenegro.

About 1,000 km^2 ^of these mountains belong to the Kosovo territory. The Albanian Alps system consists of 24 groups of mountains with 152 peaks higher than 2,000 m a.s.l. (the highest altitude in the Kosovo territory is reached by Maja e Gjeravicës at 2,460 m a.s.l.), with a large number of gorges, canyons, valleys, which make them among the most inaccessible [16], but also magnificent areas of the Balkans [17].

Due to the rich levels of biodiversity characteristic to this region, three national parks were established in the past in the Albanian Alps: one in Montenegro (Prokletije National Park) and two others in Albania (Theth and Valbona National Parks). A fourth national park in the area has been proposed to be located in Kosovo. Furthermore, Kosovo, Albania, and Montenegro are planning to join these parks and to create the cross-border Balkan Peace Park [18].

In general, Kosovo is characterised by a continental climate and in higher altitudes it is influenced by Alpine features [19]; for this reason, it has cold winters and hot summers, with an average temperature of 11.4°C. The Alpine area of Kosovo is characterised by total annual precipitation levels exceeding 2,000 mm. Specific geo-morphological, soil and climatic features provide an interesting richness and diversity of plant life in the Albanian Alps massif, with a flora belonging to three different bio-geographic zones: the Mediterranean, the Central-European and the Central-South European regions [17,20-22].

These unique features are reflected in the high plant biodiversity, which includes 1,609 taxa and ca. 150 vegetation units [23]. The most representative vegetation unites are: oriental hornbeam forest (*Carpinetum orientalis scardicu*), hop hornbeam mixed and with oriental hornbeam forest (*Ostryo-Carpinion orientalis*), thermophilous oak forests community (*Quercus frainetto *Ten., *Quercetum frainetto-cerris scardicum*, and *Quercetum petraeae-cerris*), chestnut forests (*Castanetum sativae*), beech forests (*Fagetum montanum*), and pine forests (*Pinetum heldreichii typicum*, *Pinetum heldreichii thalictretum, Pinetum peucis*, and *Pinetum mughi typicum*) [22,24].

People have withstood the extreme conditions of these areas for centuries - including very harsh winters. Until very recent decades, limitations in infrastructure and communication forced local residents to be self-sufficient in the provision of their healthcare. As a result, their primary pharmacopoeia consisted of local medicinal plants.

While recent studies on the Albanian and Montenegrin sides of the Albanian Alps have reported findings on TEK of wild medicinal and food plants [10,12,13,15], no ethnobotanical surveys have been conducted thus far in Kosovo, with the exception of a very recent work carried out by our research group in the Gollak area [9], and a review on folk botanical names in diverse Albanian-speaking areas in South-Eastern and Southern Europe [25].

The aims of this study were twofold: 1) to document the ethnobotanical knowledge related to the use of local medicinal plants in the Albanian Alps region of Kosovo; and 2) to compare the recorded data with the ethnobotanical studies recently conducted in the Albanian and Montenegrin sides of the same Albanian Alps. This was done with the overarching goal in mind of elucidating the role played by cultural/ethnic components in shaping use patterns of wild medicinal plants.

## Methods

### Field study

**Figure 1:**
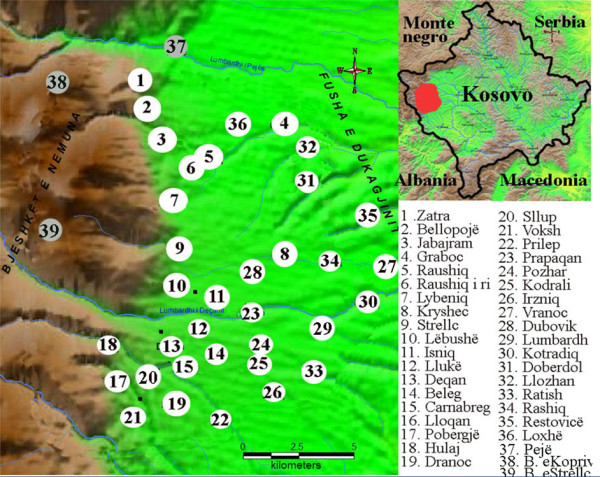
**Study area in Kosovo**.

Ethnobotanical field research was conducted in 36 villages belonging to the municipalities of Pejë and Deçan, located close to the Koprivnik and Strellc mountains, and which represent the central group of the Albanian Alps located in the western part of Kosovo (Figure 1).

The settlements and villages investigated are relatively small in terms of inhabitants (≤ 500 inhabitants per village). The native residents are ethnic Albanians, who speak Gheg varieties of the Albanian language. The exact population is currently unknown, as no population census has been conducted in Kosovo since 1981. Most recently, the area populations have been negatively affected by migration due to displacement and harsh economic conditions caused by the last Kosovo War (1998-1999).

Field studies were conducted from May to October 2010. TEK was recorded using semi-structured interviews and a questionnaire [26]. In particular, we sought the following information: respondent name and community of residence; local botanical names of useful plants; plant part(s) used; preparation/administration; local folk medicinal uses of plants.

Data were collected from 91 informants (67 male and 24 female) older than 50 years (50 to 79 years old). The respondents were mainly engaged in agricultural activities and typically inherited their ethnobotanical knowledge from their direct ancestors (parents, grandparents) via oral traditions. Study participants were selected using the snowball sampling method [2], and we particularly focused on local people who regularly use plants for medicinal purposes.

Prior informed consent was obtained conducting interviews and researchers adhered to the ethical guidelines of the International Society of Ethnobiology [27]. During the interviews, fresh plants were collected to create voucher specimens for the herbarium and the informants were followed into the field to show us the quoted species. Most plant species were collected while flowering.

Taxonomic identification was done using relevant standard botanical literature of the area [28-31]. Plant nomenclature largely follows the *Flora Europaea *[32], while plant family assignments follow the current Angiosperm Phylogeny Group guidelines [33]. Voucher specimens of the wild taxa were deposited at the Department of Biology (Herbarium code DE/10), University of Prishtina.

### Data analysis

Despite the fact that it is always problematic to compare ethnobotanical data recorded from studies conducted using different field methods and at different times, we have attempted to compare the wild medicinal plant uses recorded in Albanian Alps in Kosovo with those recorded in previously conducted ethnobotanical studies on the Albanian and Montenegrin sides of the same alpine range [11-14]. The Jaccard similarity index among the considered studies has been calculated as in the recent comparative analysis of the circum-Mediterranean medical ethnobotany [34].

## Results and Discussion

### The Kosovar medico-ethnobotany of the Albanian Alps

The results of the field survey are presented in Table 1; plants are arranged in alphabetical order by genus. For each species, the botanical name and family, local names, English name, botanical status, preparation/administration and folk medical or food uses are reported.

**Table 1:** Medicinal plant uses recorded on the Kosovar side of the Albanian Alps in the current study.

Botanical taxon, botanical family and voucher specimen code	Folk name(s) quoted by respondents	English name	Status	Quotation frequency	Part(s) used	Administration	Treated disease(s) or folk medical uses(s)
*Abies alba *Mill. (Pinaceae) 13/DE/10	Bredhi i bardhë	European silver fir	W	+	Resin	Boiled in oil	Stomach pain Eczemas
						
						Topically applied	Skin infections
						
						Mixed and boiled with milk butter	Skin hematomas Skin infections

*Achillea millefolium *L. (Asteraceae) 03/DE/10	Hajdukati	Yarrow	W	++	Areal parts	Infusion	Anti-diarrhoeal Stomach pain Anti-diabetic Eczema
						
						Tincture topical used in wound	Antibacterial

*Aconitum divergens ***Pančić (Ranunculaceae)** 04/DE/10	Pelini i egër (i zi)		W	++	Areal parts	Infusion	Stomach disorders Oral cavity antiseptic Anti-haemorrhoidal
					
					Whole plant	Infusion	Anti-cholesterolemic
					
					Leaves	Squeezed and topically applied to the wound	Anti-bacterial Skin infections

*Adiantum capillus-veneris ***L. (Adiantaceae)** 01/DE/10	Majdanozi i egër	Southern maidenhair fern	W	+	Areal parts	Decoction	Bronchitis Sour throat Expectorant

*Aesculus hippocastanum *L. (Sapindaceae) 06/DE/10	Gështenja e egër	Horse chestnut	W	++	Leaves	Infusion	Expectorant Anti-rheumatic
					
					Fruits	Decoction	Antitussive Anti-hypertensive
						
						Tincture	Anti-rheumatic

*Agropyron repens ***(L.) P. Beauv. (Poaceae)** 08/DE/10	Pirrovina	Couch grass	W	+	Roots	Decoction	Anti-rheumatic Anti-anaemic Stomach and hepatic disorders Lithontriptic
						
						Infusion	Lithontriptic

*Allium cepa *L. (Amaryllidaceae) 11/DE/10	Qepa	Onion	C	+	Leaves	Decoction	To treat influenza
					
					Bulb	Extracted with cold mineral water	Anti-hypertensive

*Allium porrum *L. (Amaryllidaceae) 09/DE/10	Purrini	Garden leek	C	+	Leaves and stem	Eaten fresh	Anti-cholesterolemic

*Allium sativum *L. (Amaryllidaceae) 10/DE/10	Hudhra	Garlic	C	+	Bulb Leaves	Tincture	Improve blood circulation Anti-diabetic Antibacterial Anti-hypertensive
						
						Decoction	Tooth ache

*Alnus glutinosa ***(L.) Gaertn. (Betulaceae)** 05/DE/10	Verri	Black alder	W	+	Cortex	Decoction, used to wash whole body	Anti-rheumatic
					
					Leaves	Extracted with cold water	Disinfectant on wounds

*Althaea officinalis *L. (Malvaceae) 07/DE/10	Mëllaga e bardhë	Marshmallow	W	++	Roots	Extracted with cold water	Expectorant
						
						Decoction	To treat lung disorders Oral cavity antiseptic Expectorant

*Arctium lappa *L. (Asteraceae) 12/DE/10	Bullushtra	Greater burdock	W	+	Areal parts	Decoction	Gastrointestinal disorders Bronchitis Lithontriptic
					Leaves	Boiled in milk (used externally	Skin inflammation and ulcers

*Aristolochia clematitis ***L. (Aristolochiaceae)** 14/DE/10	Fiku i egër	Birthwort	W	+	Fruits	Decoction	Anti-haemorrhoidal Eczemas
					
					Areal parts	Decoction	Infected wounds Ulcers

*Artemisia absinthium *L. (Asteraceae) 02/DE/10	Pelini i butë	Wormwood	W	+	Areal parts	Infusion	Stomach disorders Anti-diabetic

*Beta vulgaris ***L. (Amaranthaceae)** 17/DE/10	Sveklla	Common beet	C	+	Roots	Decoction	Anti-anaemic

*Betula verrucosa *Ehrh. (Betulaceae) 16/DE/10	Mështekna	Silver birch	W	+	Cortex	Decoction	Kidney infections
					
					Leaves	Decoction	Lithontriptic
***Brassica oleracea ***L. (Brassicaceae) 18/DE/10	Lakra	Cabbage	C	+	Leaves	Fermented leaves topically applied	Anti-bacterial

*Bryonia alba *L. (Cucurbitaceae) 15/DE/10	Stërkungulli	White bryony	W	+	Roots	Extracted with sunflower oil, apply topically in pain place	Anti-rheumatic

*Calendula officinalis *L. (Asteraceae) 28/DE/10	Lulduhani	Pot marigold	C	+	Flowers	Extracted with cold milk	Kidney disorders Hepatitis Stomach ulcers

*Capsella bursa-pastoris *(L.) Medik. (Brassicaceae) 29/DE/10	Shtrapër	Shepherd's-purse	W	+	Whole plant	Infusion	Fever Eczemas
***Capsicum annuum ***L. (Solanaceae) 32/DE/10	Speci djegës	Pepper	C	+	Fruits	Eaten fresh fruits	Anti-rheumatic Appetizing Lung disorders

*Carduus nutans *L. (Asteraceae) 27/DE/10	Gjemb gomari	Musk thistle	W	+	Inflorescences	Extracted with cold water for ten days and then used as tea	Eczemas
***Castanea sativa ***Mill. (Fagaceae) 20/DE/10	Gështenja e butë	Sweet chestnut	W/C	+	Fruits	Decoction	Headache
					
					Fruits	Decoction external applied	Anti-haemorrhoidal

*Centaurea cyanus *L. (Asteraceae) 30/DE/10	Kokoçeli	Cornflower	W	+	Flowers	Decoction	Eye infections

*Centaurium erythraea *Rafin. (Gentianaceae) 21/De/10	Kiçica	Common centaury	W	++	Areal parts	Extracted with cold water	Stomach disorders Urinary system infections
						
						Decoction	Anti-haemorrhoid Anti-diabetic Lithontriptic Fever
					
					Stem	Decoction	Lithontriptic

*Cichorium intybus *L. (Asteraceae) 22/DE/10	Çikorja	Common chicory	W	+	Stem	Infusion	Anti-diarrhoeal
					
					Roots	Decoction	Bronchitis Urinary system infections Anti-haemorrhoid
***Chelidonium majus ***L. (Papaveraceae) 31/DE/10	Tamblaqoku	Tetterwort	W	+	Areal parts	Infusion	Bronchitis Lithontriptic Stomach ulcers

*Citrullus vulgaris *Schrad. (Cucurbitaceae) 33/DE/10	Shalqiri	Watermelon	C	+	Fruit juice	Fruit juice applied into the ear	Ear-ache
					
					Seeds	Eaten dried seeds of watermelon, apple, melon	To prevent prostate cancer

*Citrus limon *(L.) Burm. f. (Rutaceae) 35/DE/10	Limoni	Lemon	C	+	Fruits	Lemon juice mixed with honey	Anti-tussive Respiratory infections

*Cornus mas *L. (Cornaceae) 24/DE/10	Thana	Dogwood	W	++	Fruits	Decoction	Anti diabetic
						
						Tincture	Stomach disorders Anti-rheumatic
						
						Consumed	Eaten raw
						
						Decoction	Anti-anaemic

*Corylus avellana *L. (Betulaceae) 25/DE/10	Lajthia	Hazel	W	+	Leaves	Decoction	Anti-diabetic

*Crataegus monogyna *Jacq. (Rosaceae) 19/DE/10	Murrizi	Oneseed	W	++	Areal parts	Infusion	Heart rhythm regulator Anti-hypertensive
					
					Fruits	Decoction	Anti-hypertensive
					
					Flowers	Decoction	Anti-hypertensive Insomnia

*Cucumis melo *L. (Cucurbitaceae) 36/DE/10	Pjepri	Melon	C	+	Seeds	Eaten dried seeds of watermelon, apple, melon	To prevent the prostate cancer

*Cucurbita pepo *L. (Cucurbitaceae) 26/DE/10	Kungulli	Pumpkin	C	+	Seeds	Eaten	Anti-helminthic To prevent prostate cancer

*Cydonia oblonga *Mill. (Rosaceae) 23/DE/10	Ftoni	Quince	C	+	Leaves	Infusion	Respiratory inflammations
					
					Seeds	Decoction	Appetizing

*Cynodon dactylon *(L.).Pers. (Poaceae) 34/DE/10	Bar magari	Bermuda grass	W	+	Roots	Decoction	Anti-haemorrhoidal
***Daucus carota ***L. (Apiaceae) 37/DE/10	Karota	Carrot	C	+	Storage root	Boiled and eaten	Stomach infections

*Digitalis grandiflora *Mill. (Plantaginaceae) 38/DE/10	Naprastak	Big-flowered foxglove	W	+	Whole plant	Infusion	Hart disorders

*Echinops bannaticus *Rochel ex Schrad. (Asteraceae) 40/DE/10	Gjembardha		W	+	Roots	Decoction	Lithontriptic

*Equisetum arvense *L. (Equisetaceae) 39/DE/10	Këputja e arave	Horsetail	W	+	Stem and Leaves	Infusion	Lithontriptic Urinary system infections

*Euphorbia cyparissias *L. (Euphorbiaceae) 41/DE/10	Bima e lythave	Cypress spurge	W	+	Stem	Fresh leaves topically applied	Warts

*Foeniculum vulgare *Mill. (Apiaceae) 43/DE/10	Kopra e egër	Fennel	W	+	Flowers	Decoction	Constipation

*Fragaria vesca *L. (Rosaceae) 42/DE/10	Dredhëza e egër	Strawberry	W	+	Leaves	Infusion	Neuro-relaxant

*Gentiana asclepiadea *L. (Gentianaceae) 45/DE/10	Utrobica		W	+	Roots	Tincture	Anti-rheumatic Stomach ulcers Hepatitis

*Gentiana lutea *L. (Gentianaceae) 44/DE/10	Sanëza		W	++	Roots	Tincture	Improve the blood circulation Bronchitis Stomach disorders Anti-hypertensive Anti-asthmatic Anti rheumatic Anti-diabetic

*Galium verum *L. (Rubiaceae) 46/DE/10	Ngjitësi i vërtetë	Yellow bedstraw	W	+	Flowers	Infusion	Urinary system infections

*Helleborus odorus *Waldst. et. Kit. (Ranunculaceae) 49/DE/10	Shpendra	Fragrant hellebore	W	+	Fruits	Applied in tooth	Tooth-ache

*Humulus lupulus *L. (Cannabaceae) 48/DE/10	Sumbullari	Common hop	W	+	Fruits	Infusion	Kidney inflammations Neuro-relaxant
					
					Areal parts	Decoction	Insomnia Menstrual cycle regulator

*Hypericum perforatum *L. (Hypericaceae) 47/DE/10	Kantarioni	St. John's wort	W	+++	Flowers	Decoction	Stomach pain
					
					Whole plant	Decoction	Respiratory disorders
					
					Areal parts	Extracted with olive oil	Stomach pain Skin infections To treat skin after sunburn or thermal burn Anti-tussive Anti haemorrhoidal Respiratory infections Anti-cholesterolemic Eczemas

*Iris *sp. (Iridaceae) 50/DE/10	Lule purriri		W	+	Leaves	Squeezed and topically applied to the ear	Ear ache

*Juglans regia *L. (Juglandaceae) 52/DE/10	Arra	Common walnut	W/C	+++	Roots	Extracted for one month with sunflower oil and then liquid mixed with honey.	Lung inflammations Anti asthmatic Bronchitis
					
					Fruits	Decoction	Anti-tussive
						
						Honey (1 kg) mixed with fruits (1 kg) extracted for one month	Lung inflammations Anti-asthmatic Anti-anaemic
						
						Extracted with cold water.	Anti-cholesterolemic
						
						Tincture	Stomach disorders
					
					Leaves	Infusion	Anti-haemorrhoid al

*Juniperus communis *L. (Cupressaceae) 51/DE/10	Gllia	Juniper	W	++	Fruits	Decoction	Back pains
						
						Extracted for 10 days in cold water mixed with lemons	Kidney inflammations Anti rheumatic
						
						Decoction	Respiratory inflammations
						
						Decoction	Stomach disorders

*Lagenaria siceraria *(Molina) Standl. (Cucurbitaceae) 53/DE/10	Pocerka	Bottle gourd	C	+	Fruits	Fruits opened and filled with water and then water used to flush the nose	Sinusitis

*Linaria peloponnesiaca *Boiss. et. Heldr. (Plantaginaceae) 57/DE/10	Lanilist		W	+	Seeds	Decoction	Constipation

*Linaria vulgaris *Mill. (Plantaginaceae) 56/DE/10	Gjineshtra	Common toadflax	W	+	Areal parts	Decoction	Urinary system inflammations

*Linum hirsutum *L. (Linaceae) 54/DE/10	Liri		W	+	Seeds	Decoction	Anti-haemorrhoidal Urinary system inflammations
					
					Leaves	Infusion	Headache Respiratory inflammations

*Lycopersicon esculentum *Mill. (Solanaceae) 55/DE10	Domatja	Tomato	C	+	Fruits	Beaked fruits mixed with sugar topically applied in wound	Wound infections

*Malus dasyphylla *Borkh. (Rosaceae) 60/DE/10	Molla sherbete	Apple	W	+	Fruits	Squeezed and topically applied to the ear	Earache

*Malus sylvestris *Mill. (Rosaceae) 61/DE10	Molla e pyllit Molla e egër	European wild apple	W	++	Areal parts	Infusion	Anti-tussive Expectorant
					
					Fruits	Extracted with cold water then fruit juice mixed sugar	Anti-hypertensive Anti-cholesterolemic
					
					Fruits	Decoction	Anti-diabetic
					
					Leaves	Applied topically in wound	Wound infections

*Matricaria recutita *L. (Asteraceae) 59/DE/10	Kamomili	Chamomile	W	++	Areal parts	Infusion	Stomachache Oral cavity inflammations Gingivitis Urinary system infections
					
					Flowers Flowers	Infusion	Oral inflammations Urinary system infections
						
						Decoction	Constipation
					
					Areal parts	Infusion	Drunk as a tea

*Melissa officinalis *L. (Lamiaceae) 58/DE/10	Bari i bletës	Lemon balm	W	+	Areal parts	Infusion	For treating abdominal pains during pregnancy
					Areal parts	Decoction	Neuro-relaxant

*Mentha longifolia *(L.) Huds. (Lamiaceae) 63/DE/10	Menta	Horse mint	W	+	Areal parts	Infusion	Neuro-relaxant Anti-diarrhoeal Anti-hypertensive

*Morus nigra *L. (Moraceae) 64/DE/10	Mani i zi	Black mulberry	W	+	Leaves	Decoction	Anti diabetic

*Origanum vulgare *L. 65/DE/10 (Lamiaceae)	Qaji i bjeshkës	Oregano	W	+	Areal parts Areal parts	Infusion	Respiratory inflammations, flu
						
						Decoction	Anti-tussive Digestive

*Orlaya grandiflora *(L.) Hoffm. (Apiaceae) 66/DE/10	Torilis	White lace flower	W	+	Areal parts	Decoction	Constipation

*Petroselinum crispum *(Mill.) Fuss (Apiaceae) 70/DE/10	Majdanozi	Parsley	C	+	Leaves	Boiled with garlic and carrot	Stomach infections
						
						Decoction together with lemon	Anti-cholesterolemic

*Pinus sylvestris *L. (Pinaceae) 69/DE/10	Çetina	Scots pine	W	++	Cones	40 cones mixed with honey (1 kg) eaten after one month	Bronchitis
						Decoction	Anti-tussive Anti-asthmatic Bronchitis

*Phaseolus vulgaris* L. (Fabaceae) 77/DE/10	Fasulja	Common bean	C	+	Seeds	2-3 soup spoons in the morning	Anti-acid

*Plantago lanceolata *L. (Plantaginaceae) 73/DE/10	Dejzi heshtor	Narrowleaf plantain	W	++	Leaves	Fresh leaves applied topically in wound	Wound infections

*Plantago major *L. (Plantaginaceae) 67/DE/10	Dejzi gjethegjerë	Common plantain	W	++	Leaves	Infusion	Back pains
						
						Eaten squeezed juice mixed with honey	Bronchitis Anti haemorrhoid Stomach-ache
						
						Applied topically in wound	Wound infections

*Polygonum bistorta *L. (Polygonaceae) 75/DE/10	Reni	Meadow bistort	W	+	Roots	Macerated roots (200-300 g) mixed honey (1 kg)	Respiratory infections Expectorant

*Populus nigra *L. (Salicaceae) 72/DE10	Plepi i zi	Black poplar	W	+	Cortex	Decoction	Urinary system inflammations
					
					Leaves	Decoction	Tuberculosis Bronchitis Anti-diabetic

*Prunus avium *(L.) L. (Rosaceae)71/DE/10	Bojlia	Wild cherry	C	+	Fruits	Infusion	Anti- diabetic Anti-hypertensive Respiratory inflammations

*Prunus domestica *L. (Rosaceae) 68/DE/10	Kumbulla	Plum	C	+	Fruits	Decoction	Constipation

*Prunus spinosa *L. (Rosaceae) 74/DE/10	Kulumria	Blackthorn	W	+	Fruits	Decoction	Anti-hypertensive Anti-asthmatic
						
						Eaten fresh fruits	Consumption

*Pteridium aquilinum *Kuhn. (Dennstaedtiaceae) 76/DE/10	Fieri	Bracken	W	+	Leaves	Decoction	Anti-bacterial Diuretic

*Pyrus pyraster *(L.) Du Roi (Rosaceae) 78/DE/10	Dardha e egër	Wild pear	W	+	Fruits	Tincture	Anti-hypertensive Anti-cholesterolemic

*Robinia pseudoacacia *L. (Fabaceae) 82/DE/10	Bagreni	Black locust	W	+	Flowers	Decoction	Respiratory inflammations

*Rosa canina *L. (Rosaceae) 80/DE/10	Kaça	Dog rose	W	+	Fruits	Infusion	Drunk as a tea
					
					Fruits	Decoction	Influenza Increase immunity

*Rubus fruticosus *L. (Rosaceae) 79/DE/10	Mani	Blackberry	W	++	Leaves	Fresh leaves applied topically in wound	Skin infection
					
					Leaves and fruits	Decoction	Tuberculosis Influenza Increase immunity
					
					Fruits	Eaten fresh fruits Jam	Consumption

*Rubus idaeus *L. (Rosaceae) 80/DE/10	Mjedra	Raspberry	W	+	Leaves	Decoction	Sore throat Influenza Increase immunity

*Sambucus ebulus *L. (Adoxaceae) 83/DE/10	Kinla	Dwarf elderberry	W	++	Areal parts	Topically in applied in pain place	Anti rheumatic
					
					Fruits	Tincture	Menstrual pains Regulation of menstrual cycle
					
					Flowers	Tincture	Urinary inflammations

*Sambucus nigra *L. (Adoxaceae) 85/DE/10	Shtogu	Elderberry	W	+++	Stem cortex	Extracted with sunflower oil	To treat sunburns
						
						Boiled with butter milk	To treat thermal burns
					
					Flowers	Infusion mixed with lemon and sugar	Anti asthmatic Bronchitis
						
						Infusion	Antitussive
					
					Fruits	Drunk fruit juice	Anti-anaemic
					Areal parts	Decoction	Anti-allergic

*Salix purpurea *L. (Salicaceae) 86/DE/10	Shelgu	Purple willow	W	+	Leaves	Applied topically in breast	Anti-fever

*Salvia officinalis *L. (Lamiaceae) 88/DE/10	Sherbela	Garden sage	C	+	Leaves	Decoction	Sedative Antipyretic

*Sempervivum tectorum *L. (Crassulaceae) 87/DE/10	Bar veshi	Houseleek	W	+	Leaves	Decoction after cooled applied in ear	Ear ache

*Solanum tuberosum *L. (Solanaceae) 84/DE/10	Patatja	Potato	C	+	Tuber	Cut in several pieces and placed in front of the head	Head-ache

*Taraxacum officinale *F.H. Wigg. (Asteraceae) 96/DE/10	Lule dielli	Dandelion	W	+	Flowers	Decoction mixed with lemon fruits.	Bronchitis

*Teucrium chamaedrys *L. (Lamiaceae) 94/DE/10	Arrsi i vogël	Wall germander	W	+	Areal parts	Infusion	Anti-haemorrhoidal
					
					Whole parts	Infusion	Anti diabetic

*Thymus *spp. (Lamiaceae) 93/DE/10	Shpirti i nënës	Wild thyme	W	++	Areal parts	Decoction	Respiratory inflammations Expectorant
					
					Whole plant	Infusion	Bronchitis Anti-tussive Expectorant
					
					Areal parts	Infusion	Lung inflammations Expectorant

*Tilia platyphyllos *Scop. (Malvaceae) 95/DE/10	Blini	Largeleaf linden	W	+	Flowers	Decoction	Sore throat Lung inflammations

*Trifolium pratense *L. (Fabaceae) 92/DE/10	Tërfoja e kuqe	Red clover	W/C	+	Leaves	Squeezed leaves juice	Stomach disorders

*Trifolium repens *L. (Fabaceae) 91/DE/10	Tërfili i bardhë	White clover	W	+	Flowers	Decoction	Anti-diarrhoeal

*Triticum vulgare *L. (Poaceae) 89/DE/10	Gruri Karajpeli	Wheat	C	+	Seeds	Boiled seeds with water and added sugar	Constipation Anti-haemorrhoid
					
					Flowers	Decoction	Kidney disorders Anti rheumatic Neuro-relaxant

*Urtica dioica *L. (Urticaceae) 97/DE/10	Hithi	Common nettle	W	++	Leaves	Eaten fresh	Anti anaemic
					
					Leaves and stem	Tincture	Improve blood circulation
					
					Roots and Leaves	Decoction	Alopecia
					
					Roots	Decoction	Anti-haemorrhoidal

*Vaccinium myrtillus *L. (Ericaceae) 98/DE/10	Boronica	Bilberry	W	+	Areal parts	Infusion	Anti-diabetic
					
					Fruits and Leaves	Decoction	Neuro-relaxant Urinary inflammations Lung inflammations Stomach disorders Anti-hypertensive
					
					Fruits	Eaten fresh	Consumed

*Veratrum album *L. (Melanthiaceae) 99/DE/10	Shtara	White hellebore	W	+	Leaves	Decoction	Anti-lice
					
					Roots	Decoction	Head ache
					
					Leaves	Extracted with sunflower oil	Eczemas Haemorrhoids

*Zea mays *L. (Poaceae) 100/DE/10	Misri	Corn	W	+	Silks	Infusion	Anti-diabetic

+ quoted by less than 5% of the participants; ++ quoted by more than 5% and less than 30% of the participants; +++ quoted by more than 30% of the participants

We found that 98 species (belonging to 39 families) are employed in the traditional medicine of the area. These includes three fern species, three gymnosperms and 92 angiosperms (84 dicotyledonous and 8 monocotyledons); 74 taxa are wild. Of these species, *Achillea millefolium *L., *Cornus mas *L., *Hypericum perforatum *L., *Juglans regia *L., *Juniperus communis *L., *Malus sylvestris *Mill., *Plantago major *L., *Sambucus nigra *L. were cited more then 30% of the informants. From 98 species presented in Table 1, 23 species are also included in the official Pharmacopoeia of Europe [35].

The predominantly quoted botanical families were Rosaceae (12%), Asteraceae (10%), and Lamiaceae (5%). These same three "top" families were found to be also predominant among the wild medicinal taxa used in the folk medicine of the Alps in Montenegro, Albania, and in the Gollak region in Kosovo [9,11-14].

The most frequently quoted manner of preparation of medicinal plants was represented by decoctions (51%) and infusions (26%). The most frequently cited medicinal uses referred to gastrointestinal (26%), respiratory (19%) troubles, and illnesses affecting the urogenital system (12%). The first two categories were also the most frequently quoted in the ethnobotanical studies conducted on the Montenegrin and Albanian sides [11-14].

### Most uncommon medicinal plants

Upon analysis of the bio-pharmacological literature on the quoted medicinal species available on PubMed, we found that it could be worthwhile to further investigate the following reports:

1. The internal use of cold water macerates of the inflorescences of *Carduus nutans *L. in the treatment of eczema (this taxon is scarcely known in the phytochemical and pharmacological literature). In 2000 a Turkish research group pointed out the hepatoprotective effects of extracts from this plant [36];

2. The internal use of decoction of the roots of *Echinops bannaticus *Rochel ex Schrad. for kidney stones (despite a few studies on other species of the genus *Echinops*, this Balkan species is largely under-investigated); and

3. The internal use of decoctions of aerial parts of *Orlaya grandiflora *Hoffm. for its laxative effects (the plant is completely unknown in the phytopharmacological literature).

### Comparison with the Albanian and Montenegrin Alpine ethnobotanical literature

**Figure 2:**
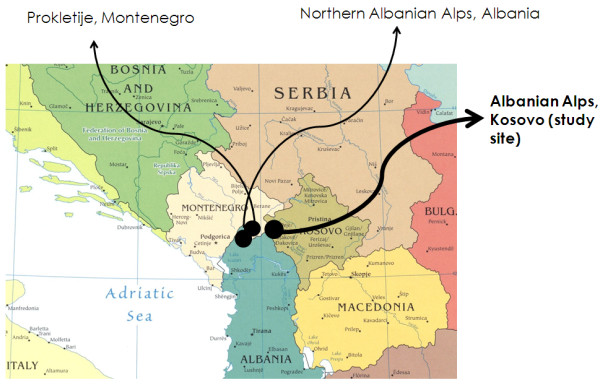
**Location of the study area in Kosovo and of the sites where previous ethnobotanical works have been conducted in Albania and Montenegro **[11-14] .

Table 2 and Figure 2 show the sites and field studies that have been compared with the data gathered in the Kosovar Alps.

**Table 2:** Summary of the field ethnobotanical studies considered in the cross-cultural data analysis.

Area and country	Study participants	Year(s) when the field studies were conducted	Number of study participants	Reference(s)
Albanian Alps (Kosovo)	Albanians	2010	91	Current study
Prokletije mountains (Montenegro)	Bosniaks and Serbs	2006 and 2007	75	[15]
Northern Albanian Alps (Albania)	Albanians	2004, 2005, and 2007	62	[11-14]

**Figure 3:**
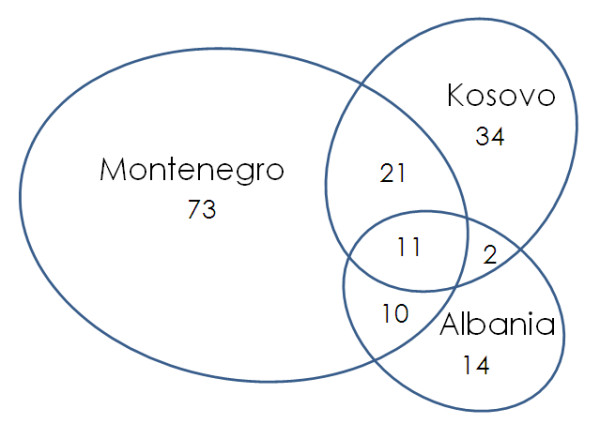
**Representation of the commonalities among the wild medicinal species quoted on the Kosovar, Montenegrin, and Albanian sides of the Albanian Alps [data from the current study and **[11-14]**]**.

Figure 3 and Table 3 illustrate the similarity between the wild medicinal plants used and recorded in the current study and those recorded in the Montenegrin and Albanian sides of the same Albanian Alps.

**Table 3:** Jaccard similarity index of the wild medicinal plants used in the Kosovar, Albanian, and Montenegrin Alps.

Group I	Group 2	Species used in both groups	Species used in one group only (Group 1/Group 2)	Jaccard Index
Albanians in Albania	Albanians in Kosovo	13	24/45	15.9
Albanians in Albania	Serbs and Bosniaks in Montenegro	21	16/94	16.0
Serbs and Bosniaks in Montenegro	Albanians in Kosovo	32	83/36	21.2

The link between the medical ethnobotany of the Montenegrin and Kosovar sides of the Alps - despite the different ethnicity/language of the local populations - appears stronger than the link between the ethnobotany of these two locations and the ethnobotany Albania.

This apparent paradox could be explained in a number of ways:

1. Different sampling techniques may have been adopted during the field survey in the three locations or the socio-economic background of the interviewees could have been different. For example, on the Albanian side of the Alps, the previous ethnobotanical studies selected local informants from very remote areas, which remained quite isolated during Communist times and with very limited access to urban environments and culture. It could be especially worthwhile to further assess the influence of the popular phytotherapeutical literature on folk medicine in Montenegro and Kosovo, since during the Yugoslavian time this kind of popularised knowledge was said to be "en-vogue". For example, this is very evident in the Montenegrin data, where a number of possible "modern" uses of local medicinal plants (i.e. *Hypericum perforatum *used as an anti-depressive) were recorded.

2. The study sites chosen in Kosovo and Montenegro are on average located at lower elevations than the sites selected in Northern Albania, thus resulting in a partially different ecological setting and availability of certain species in the environments.

3. Both the Montenegrin and Kosovar side of the Albanian Alps have had a common history for the most part of the 20th Century, since belonging to the same country (former Yugoslavia). This may have "homogenised" eventual pre-existing differences in plant perceptions/uses between the Albanian and Slav communities. Moreover, a few South-Slav communities (i.e. Bosniaks [2-4,7,8]) could be surely considered much more "herbophilic" than the Albanian ones, and this may have influenced the folk medicine of the Kosovar population to a certain degree during the last century, who have always lived in contacts with the Slavs.

4. The Montenegrin study included self-declaring Serbian and Bosniak communities. However, a large part of the Bosniak community living in the Gusinje area is represented also by "bosniakised" Albanians, whose Catholic tribes settled on this side of the Albanian Alps and converted to Islam a couple of centuries ago [37]. This could mean that the ethnobotanical data of Montenegro and Kosovo may actually refer to the same core of Muslim Albanians.

Despite the commonalities found on the quoted medicinal plants, Table 4 shows the different uses of the wild taxa, which have been most quoted in all three sides of the Alps.

**Table 4:** Comparison of the most quoted folk medicinal uses of wild taxa in the current study and in ethnobotanical studies previously conducted in Albania and Montenegro [11-14] (Same or similar uses are underlined

Botanical taxon	Used part(s)	Pathologies treated in the folk medicine of the Kosovar Alps	**Pathologies treated in the folk medicine of the Montenegrin Alps **[15]	**Pathologies treated in the folk medicine of Albanian Alps **[11-14]
*Centaurium erythraea *Rafn.	Aerial parts	Stomach-disorders Diabetes Fever Kidney stones and UTIs	Stomach disorders and loss of appetite Diabetes	Fever
*Cornus mas *L.	Fruits	Stomach disorders Diabetes Rheumatisms Anaemia	Dhiarroea	Intestinal troubles
*Gentiana *spp.	Roots	Blood circulation- related diseases (including hypertension) Bronchitis and asthma Stomach disorders Rheumatisms	Stomach-ache	Cardiovascular diseases
*Hypericum *spp.	Flowering aerial parts	Stomach disorders Bronchitis and asthma Hypertension Skin infections, sunburns, and eczemas Haemorrhoids Anti-cholesterolemic	Gastritis Anxiety and depression Skin inflammations and burns	Stomach and digestive disorders Anxiety Respiratory diseases Fever UTIs
*Origanum vulgare *L.	Aerial parts	Respiratory diseases Digestion UTIs	Respiratory diseases Digestive	Respiratory diseases Digestive Diuretic
*Plantago *spp.	Aerial parts	Stomach-ache Respiratory diseases Wounds Haemorrhoids Back-pains	Respiratory diseases Mouth and skin inflammations Fever Haemorrhoids	Abdominal pains Wounds Diuretic
*Urtica dioica *L.	Roots	Haemorrhoids Alopecia	Haemorrhoids Fever Arthritis Anaemia Alopecia UTIs	Rheumatisms Alopecia Genital problems

UTIs: Urinary Tract Infections

From Table 4 it is interesting to underline that the folk uses of the wild medicinal taxa recorded in Kosovo often include both the uses recorded in Albania and those in Montenegro. It would then appear that the medico-ethnobotany of Kosovo - because of its history in the last century and the exposure to the South-Slavic ethnobotanical traditions - has possibly incorporated both Albanian and Slavic plant uses.

## Conclusions

Medicinal plants still play a crucial role in the sphere of human health in the Albanian Alps, not only in the Montenegrin and Albanian territory, but also on the Kosovar side. Oftentimes, these mountainous communities have limited or non-existent access to Western biomedical modalities, and are instead self-reliant on their TEK. The local flora is thus incredibly important to provide the first health care within the households of the Albanian Alps.

Moreover, the biodiversity richness and unique bio-cultural heritage of the local people here is something to be highly valued. Steps towards this end are evident in the formation of protected parks for biodiversity conservation - but further efforts in conservation of the human TEK diversity and cultural heritage are necessary as well. TEK-dependent activities such as sustainable gathering of wild medicinal taxa, their small-scale trade, and production of local high quality plant-based foods and dairy products can all contribute to the growing eco-tourism initiatives. Thus, TEK is a critical component to success in the future economic development and biocultural conservation efforts of the region.

## Competing interests

The authors declare that they have no competing interests.

## Authors' contributions

BM and AH conceived the study, and participated in its design and coordination. AH and HA carried out the field study; EH and FK verified the identification of the plant taxa; AH, AP, and CLQ performed the data analysis and drafted the discussion. CLQ edited the manuscript. All authors read and approved the final manuscript.
